# Role of Oxidative Stress in the Pathogenesis of Atherothrombotic Diseases

**DOI:** 10.3390/antiox11071408

**Published:** 2022-07-20

**Authors:** Giovanna Petrucci, Alessandro Rizzi, Duaa Hatem, Giulia Tosti, Bianca Rocca, Dario Pitocco

**Affiliations:** 1 Department of Bioethics and Safety, Section of Pharmacology, Catholic University School of Medicine, 00168 Rome, Italy; giovanna.petrucci@unicatt.it (G.P.); duaa.hatem@unicatt.it (D.H.); 2 Fondazione Policlinico Universitario A. Gemelli IRCCS, 00168 Rome, Italy; 3 Diabetes Care Unit, Fondazione Policlinico Universitario A. Gemelli IRCCS, 00168 Rome, Italy; alessandro.rizzi@unicatt.it (A.R.); giulia.tosti93@gmail.com (G.T.); dario.pitocco@unicatt.it (D.P.); 4 Catholic University School of Medicine, 00168 Rome, Italy

**Keywords:** reactive oxygen species, oxidative stress, atherosclerosis, cardiovascular diseases, platelets, scavengers, biomarkers, antioxidants

## Abstract

Oxidative stress is generated by the imbalance between reactive oxygen species (ROS) formation and antioxidant scavenger system’s activity. Increased ROS, such as superoxide anion, hydrogen peroxide, hydroxyl radical and peroxynitrite, likely contribute to the development and complications of atherosclerotic cardiovascular diseases (ASCVD). In genetically modified mouse models of atherosclerosis, the overexpression of ROS-generating enzymes and uncontrolled ROS formation appear to be associated with accelerated atherosclerosis. Conversely, the overexpression of ROS scavenger systems reduces or stabilizes atherosclerotic lesions, depending on the genetic background of the mouse model. In humans, higher levels of circulating biomarkers derived from the oxidation of lipids (8-epi-prostaglandin F_2α_, and malondialdehyde), as well as proteins (oxidized low-density lipoprotein, nitrotyrosine, protein carbonyls, advanced glycation end-products), are increased in conditions of high cardiovascular risk or overt ASCVD, and some oxidation biomarkers have been reported as independent predictors of ASCVD in large observational cohorts. In animal models, antioxidant supplementation with melatonin, resveratrol, Vitamin E, stevioside, acacetin and n-polyunsaturated fatty acids reduced ROS and attenuated atherosclerotic lesions. However, in humans, evidence from large, placebo-controlled, randomized trials or prospective studies failed to show any athero-protective effect of antioxidant supplementation with different compounds in different CV settings. However, the chronic consumption of diets known to be rich in antioxidant compounds (e.g., Mediterranean and high-fish diet), has shown to reduce ASCVD over decades. Future studies are needed to fill the gap between the data and targets derived from studies in animals and their pathogenetic and therapeutic significance in human ASCVD.

## 1. Introduction

**Figure 1:**
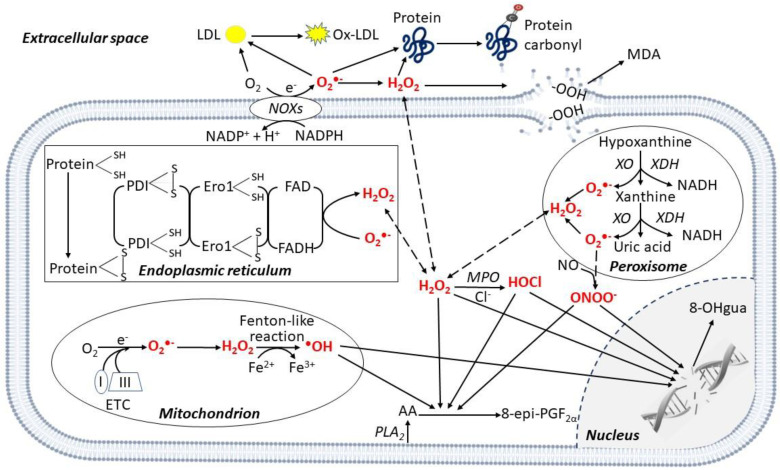
Enzymatic and non-enzymatic production of reactive oxygen species in different cell compartments. Reactive oxygen species (ROS) are produced in different cellular compartments. Mitochondria generate a high quantity of ROS through the electron transport chain (ETC), mainly complexes I and III, and the ^•^OH is produced via the Fenton-like reaction. Other ROS-producing mechanisms involve transmembrane nicotinamide adenine dinucleotide phosphate (NADPH) oxidases (NOXs), xanthine oxidase (XO) in peroxisomes, and protein disulfide isomerase (PDI) in the endoplasmic reticulum. ROS oxidize polyunsaturated lipids from membranes releasing 8-epi-prostaglandin F_2α_ (8-epi-PGF_2α_) from arachidonic acid (AA), and malondialdehyde (MDA). In the cytoplasm, myeloperoxidase (MPO) mediates HOCl formation from Cl^−^. In the nucleus, ROS induce DNA damage, releasing 8-hydroxy-2′-deoxyguanosine (8-OHgua). In the extracellular space, ROS mediate the oxidation of proteins, generating protein carbonylation. Specifically, in the peripheral blood the oxidation of low-density lipoprotein (LDL) generates oxidized (ox)-LDL. Abbreviations: Ero1: Endoplasmic Reticulum Oxireductin 1; FAD: Flavin Adenine Dinucleotide; PLA_2_: Phospholipase A_2_; XDH: Xanthine Dehydrogenase.

Oxidative stress is generally defined as an imbalance between formation of reactive oxygen species (ROS) [1] and their clearance by antioxidant systems [2]. ROS include molecules and free radicals (i.e., chemical species with one unpaired electron) derived from molecular oxygen (O_2_) formed in the cell cytoplasm, endoplasmic reticulum (ER), mitochondria, peroxisomes [3,4] and extracellular space (Figure 1).

While O_2_ by itself is not very reactive, if one of its unpaired electrons is excited, the resulting species become powerful oxidants [5]. Superoxide anion (O_2_^•^^−^), is the precursor of most ROS, such as hydrogen peroxide (H_2_O_2_), which may then generate the hydroxyl radical (^•^OH) and the peroxynitrite (ONOO^−^) by reacting with nitric oxide (NO) [4] (Figure 1). O_2_^•−^ can be produced during enzymatic reactions, e.g., by cytochrome P450, nicotinamide adenine dinucleotide phosphate (NADPH) oxidases (NOXs), or xanthine oxidase (XO) in the cell cytoplasm [2]. O_2_^•−^ can also be non-enzymatically released along the mitochondrial electron transport chain (ETC) reactions, especially by complexes I and III [3,4] (Figure 1). Depending on their origin, type and environment, ROS-triggered signals may contribute to both cell homeostasis [3,6] or dysfunction by the non-specific damage of proteins, lipids, nucleic acids, and polysaccharides [4].

**Figure 2:**
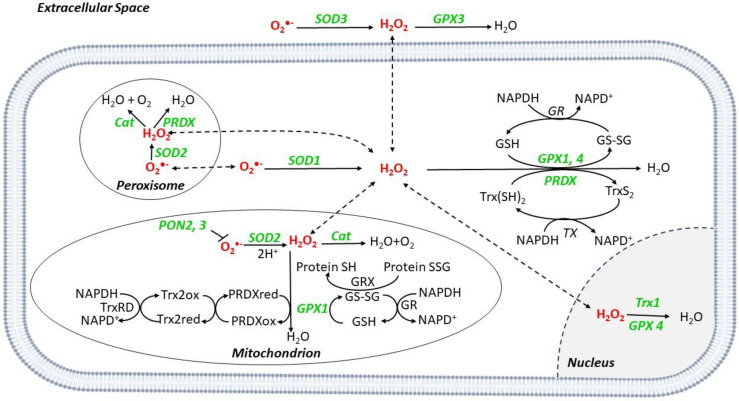
ROS scavenger systems in different cell compartments. O_2_^•−^ is converted to H_2_O_2_ by superoxide dismutases (SODs), SOD1 in the cytoplasm, SOD2 in the mitochondria, and peroxisome, and SOD3 in the extracellular space. Catalase (Cat) catalyzes the reduction from H_2_O_2_ to O_2_ and H_2_O in mitochondria and peroxisome. Glutathione peroxidases (GPX) catalyze the reduction in H_2_O_2_; during the reaction, glutathione (GSH) is converted to its oxidized form (GSSG), which has a decreased ability to reduce peroxide. Once oxidized, GSH can be regenerated from GSSG by the enzyme glutathione reductase (GR) using reduced nicotinamide NADPH as the electron donor. During the process, NADPH is oxidized to NADP^+^. Peroxiredoxins (PRDX) reduce H_2_O_2_ to H_2_O by utilizing electrons from NADPH via thioredoxin (Trx) and thioredoxin reductase (TR). Paraoxonase (PON) isoforms 2 and 3 can prevent mitochondrial O_2_^•−^ generation. Abbreviations: GRX: Glutaredoxin; XO: Xanthine Oxidase.

The main ROS-buffering systems in the mitochondria include glutaredoxin (GRX), glutathione (GSH) and thioredoxin (Trx) [7]. Superoxide dismutases (SOD) are metalloenzymes converting O_2_^•−^ into H_2_O_2_, which can be then degraded into H_2_O and O_2_ by the GSH redox system that includes glutathione reductases (GR), glutathione peroxidase (GPX), and peroxiredoxins (PRDXs) [8] (Figure 2).

SOD2 is mitochondrial, while SOD1 and 3 are cytoplasmic and extracellular, respectively [8]. Catalase (Cat) is a peroxisome scavenger enzyme, converting H_2_O_2_ into H_2_O and O_2_ [8] (Figure 2).

**Figure 3:**
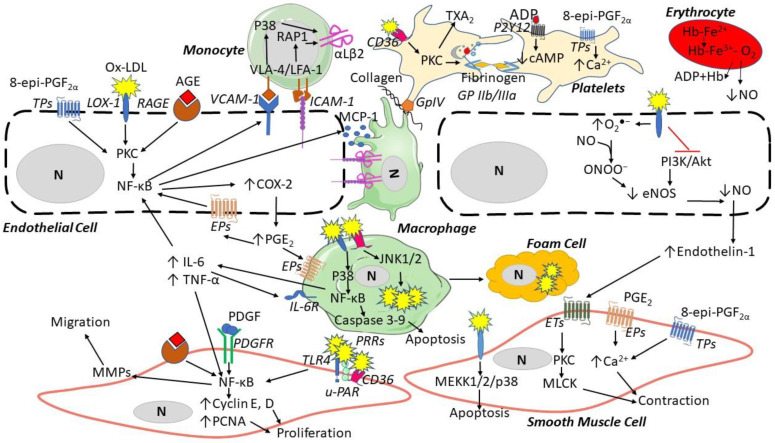
ROS contribution to the formation of atherosclerotic lesions. Oxidized low-density lipoproteins (ox-LDL) and advanced glycation end-products (AGEs) can bind their receptors (LOX-1 and RAGE, respectively) and induce endothelial cell dysfunction by increasing the expression of vascular adhesion molecule-1 (VCAM-1), intracellular adhesion molecule (ICAM-1), inducing the secretion of monocyte chemotactic protein-1 (MCP-1), and reducing nitric oxide. Endothelial dysfunction then induces monocyte adhesion, the expression of αLβ2 integrin binding ICAM-1, migration to the media, and differentiation into macrophages, which then release inflammatory cytokines (e.g., interleukin(IL)-6 and tumor necrosis factor-alpha (TNF-α)). Platelets are activated by Ox-LDL through CD36 binding and 8-epi-PGF_2α_ triggers platelet aggregation via thromboxane (TX) A_2_ receptors (TP), releasing adenosine diphosphate (ADP). In smooth muscle cells, ox-LDL via LOX-1, AGEs via RAGE, platelet-derived growth factor (PDGF), and endothelin-1 can induce proliferation, apoptosis, and contraction through several pathways. Abbreviations: Akt: protein kinase B; CD36: cluster of differentiation 36; COX: cyclooxygenase; eNOS: endothelial nitric oxide synthase; EPs: prostaglandin E_2_ receptors; ET: endothelin receptor; Hb: hemoglobin; JNK: c-Jun N-terminal kinase; LOX: lectin-like oxidized LDL receptor; LFA: lymphocyte function-associated antigen; MEKK: mitogen-activated protein kinase kinase; MMPs: matrix metalloproteinases; MLCK: myosin light-chain kinase; N: nucleus; PI3K: phosphatidylinositol 3-kinase; PAR: protease-activated receptor; PDGFR: platelet-derived growth factor receptor; PGE_2_: prostaglandin E_2_; NF-κB: nuclear factor-kappa; PCNA: proliferating cell nuclear antigen; P2Y: purinergic receptor; PKC: protein kinase C; p38: mitogen-activated protein kinases; PRRs: pattern recognition receptors; RAGE: receptors of advanced glycation end products; TLR: toll-like receptor; u-PAR: urokinase plasminogen activator receptor; VLA: vascular leukocyte adhesion molecule.

Several pre-clinical data suggest that ROS contribute to atherosclerosis through endothelial cell (EC) dysfunction, platelet activation and vascular remodeling [9] (Figure 3), while the translation of pre-clinical evidence into human atherosclerotic cardiovascular disease (ASCVD) seems more complex and less clear. The present review will revise pre-clinical, clinical and intervention evidence of ROS involvement in atherosclerosis development and its thrombotic complications.

## 2. ROS Generation

Animal models supporting the contribution of ROS in atherosclerosis are summarized in Table 1.

NOX isoenzymes transport electrons across biological membranes, reducing O_2_ into O_2_^•−^ (Figure 1), and consist of seven isoforms: NOX1 to 5, and dual oxidase 1 and 2 [34]. NOX1, 2, and 4 have been extensively investigated as ROS generators in mouse models. NOX1, which is mainly expressed in rodent’s ECs and vascular smooth muscle cells (VSMCs) [35], can be activated by different pro-thrombotic stimuli, including angiotensin II (AngII), and platelet-derived growth factor (PDGF) [36]. In apolipoprotein (Apo)E knockout (^−/−^) mice that develop a disease similar to human atherosclerosis, NOX activator-1 is increased in aortic atherosclerotic lesions [10], and double ApoE^−/−^/NOX1^−/−^ mice show reduced O_2_^•−^ in the heart and atherosclerotic lesions vs. ApoE^−/−^ animals [3,12,13] (Table 1). In coronary arteries isolated from transplanted hearts, O_2_^•−^ is higher in the coronary artery with atherosclerosis [27]. However, low NOX1 expression has also been reported in atherosclerotic coronary arteries of patients undergoing bypass grafting while NOX2 and NOX4 expression were significantly higher in vessels with coronary artery disease (CAD) vs. non-CAD [27,28] (Table 1); thus, whether NOX1 is involved in human atherogenesis is unclear.

NOX2 is highly expressed in rodent’s ECs, fibroblasts, and VSMCs [35,37], and can be activated by AngII, thrombin, endothelin, tumor necrosis factor-α (TNF-α), interleukin (IL)-1, PDGF [38,39,40]. In ApoE^−/−^ mice, NOX2 is upregulated in aortic ECs with early vascular lesions [11] (Table 1). High-fat diet (HFD)-fed ApoE^−/−^ mice treated with an NOX2 inhibitor show reduced O_2_^•−^ in aortic lesions [14], NOX2^−/−^ mice are protected from injury-induced neointima formation [15] and show poor platelet adhesion to injured arteries [41], double ApoE^−/−^/NOX2^−/−^ mice show reduced aortic O_2_^•−^ levels and atherosclerosis [11] (Table 1). EC-targeted NOX2 overexpression (^+/+^) in ApoE^−/−^ mice increases O_2_^•−^ levels and macrophage infiltration into early atherosclerotic lesions [17]. NOX2 requires activation through interactions between subunits, including p47 phagocyte oxidase (phox) and gp91 phox [42]. ApoE^−/−^ p47 phox^−/−^ or gp91 phox^−/−^ mice have significantly decreased atherosclerosis, O_2_^•−^ and increased NO in the aortas vs. ApoE^−/−^ mice [11,18] (Table 1). Triple-NOX1^−/−^/NOX2^−/−^/NOX4^−/−^ mice show reduced platelet O_2_^•−^ formation and aggregation [19], (Table 1). In humans, congenital NOX2 deficiency is associated with a rare X-linked chronic granulomatous disease (CGD) [29]. This defect is characterized by low atherosclerosis [29], circulating oxidized low-density lipoprotein (ox-LDL), urinary 8-epi-prostaglandin F_2α_ (8-epi-PGF_2α_), a non-enzymatic product of lipid oxidation [29], low O_2_^•−^ and high NO generation from platelets [43] (Table 1), suggesting a role for NOX2 in human atherogenesis.

NOX4 is expressed in rodent’s VSMCs, fibroblasts, and ECs [16]. It produces H_2_O_2_ via its E-loop, accelerating O_2_^•−^ dismutation [44] (Figure 1). ApoE^−/−^/LDL receptor (LDLr)^−/−^ mice show increased NOX4 in aortic lesions and NOX4 knockdown, with short interfering (si)RNA in the aortic SMCs of these mice decreases H_2_O_2_, suggesting a role for NOX4-derived cellular ROS in atherosclerosis [20]. In humans, NOX4 is expressed in VSMCs, ECs and fibroblasts [45,46]. The in vitro NOX4 depletion of human umbilical vein EC (HUVECs) using small-hairpin RNA, decreases O_2_^•−^ and 8-oxodeoxyguanosine, a marker of oxidative DNA damage [47] (Table 1). Human macrophages isolated from healthy individuals and treated with ox-LDL upregulate NOX4, increase O_2_^•−^ and H_2_O_2_ and undergo death [48,49]. NOX4 expression in coronary artery intima increases with atherosclerosis severity in transplanted hearts [50]. 

Myeloperoxidase (MPO) is a leukocyte enzyme that generates hypochlorous acid (HOCl) [32,51]. HFD-fed ApoE^−/−^ mice irradiated and reconstituted with MPO^−/−^ bone marrow show reduced atherosclerosis [24]; MPO^−/−^ mice and wild-type WT mice treated with an MPO inhibitor show reduced neointima formation following ischemia reperfusion (I/R) injury [24] (Table 1). However, LDLr^−/−^ mice irradiated and reconstituted with bone marrow MPO^−/−^ exhibit a significant increase in aortic atherosclerotic lesions vs. LDLr^−/−^ mice [25] (Table 1). Human atherosclerotic thoracic arteries express higher MPO levels than normal ones [33] (Table 1), and MPO was significantly increased in the coronary atherosclerotic lesion of transplanted hearts [32] (Table 1).

Mitochondrial dysfunction in the cell can generate a disproportionate O_2_^•−^ rate [52], which may damage mitochondrial DNA (mtDNA) [53]. Consistently in early aortic atherosclerotic lesions of ApoE^−/−^ mice, mtDNA integrity is decreased, O_2_^•−^ is increased, and treatment with a mitochondrion-targeted antioxidant significantly reduces H_2_O_2_ and atherosclerosis [54,55]. Protein kinase R-like endoplasmic reticulum resident kinase (PEAK) is a stress-sensor protein that decreases translation in response to stress [56]. In a rat cardiomyoblast cell line, PEAK silencing increases mitochondrial activity and ROS, while cardiac PEAK^+/+^ mice seem to be protected from I/R injury and show a significant decreased mitochondrial complex I activity [57].

MicroRNA-210 regulates cellular hypoxia response by targeting the mitochondrial energy metabolism [58]. In MicroRNA-210^−/−^ mice, mitochondrial ROS significantly increase after I/R vs. WT [26] (Table 1). In humans, atherosclerotic carotid endarterectomies show a lower mtDNA copy number than normal vessels [59].

In conclusion, genetically modified animal models show that several enzymatic and non-enzymatic reactions that generate ROS can contribute to different phases of atherosclerosis. Human evidence on the same enzymes is more limited and often inconsistent.

## 3. Scavenger Systems

Studies on ROS scavenger systems are summarized in Table 2.

Cat is localized in the peroxisomes that are adjacent to the mitochondria (Figure 2) of mammalian tissues [8,94]. In LDLr^−/−^ mice on HFD, mitochondrial O_2_^•−^ suppression in macrophages through mitochondrial Cat overexpression is associated with reduced aortic atherosclerosis [3]. Mitochondrial oxidative stress appears to be reduced by Cat^+/+^ targeted to macrophages or myeloid cells in LDL^−/−^ mice, with reduced aortic lesions [63,64], and ApoE^−/−^/Cat^+/+^ mice show reduced aortic atherosclerosis and 8-epi-PGF_2α_ expression [60,61] (Table 2). Cat^+/+^ in aortic VSMCs reduces apoptosis through TNF-α and metalloproteases reduction in mice [62]. Interestingly, adenovirus-mediated Cat^+/+^ in human aortic ECs in vitro reduces ox-LDL-induced O_2_^•−^ and apoptosis via Jun N-terminal kinase inhibition and extracellular signal-regulated kinase phosphorylation, which are downstream effectors of mitogen activator protein kinase [95], with the latter being involved in atherosclerosis development in mice. In humans, Cat gene mutations cause Acatalasemia, which is characterized by low Cat levels, diabetes mellitus (DM), and increased atherosclerosis [82] which are hypothesized to be secondary to H_2_O_2_ increase [83,84]. 

GPXs are selenoproteins that catalyze the reduction in H_2_O_2_ and other peroxides (e.g., lipids of the cell membrane) using GSH as a substrate [96] (Figure 2). They include cytosolic and mitochondrial GPX1, extracellular GPX3, and GPX4 expressed in the cytosol, mitochondria, and nucleus [97]. In hyperhomocysteinemic cystathionine beta-synthase-deficient mice, GPX1^+/+^ restores normal, EC-dependent vascular function [98] (Table 2). Consistently, in ApoE^−/−^/GPX1^+/+^ mice, atherosclerotic lesions and aortic 8-epi-PGF_2α_ content are reduced [68], while the opposite occurs in ApoE^−/−^/GPX1^−/−^ mice [70,71] (Table 2). GPX1^+/-^ mice show increased mesenteric vasoconstriction, perivascular matrix deposition, and plasma 8-epi-PGF_2α_ [69]. The upregulation of GPX1 in human ECs in vitro decreases the expression of proatherogenic genes such as CD40, monocyte chemoattractant protein-1 (MCP-1), and vascular cell adhesion protein-1 (VCAM-1) [86] (Table 2). In 101 patients undergoing coronary stenting, erythrocytes with the 599C/T allele of the GPX1 gene exhibit low activity of GPX, ox-LDL, and a higher risk of restenosis vs. WT allele homozygotes [85] (Table 2). Other studies show that GPX1 activity in washed erythrocytes is inversely correlated with CAD and acute myocardial infarction (MI) [99,100,101].

The paraoxonase (PON) proteins seem be antioxidant by hydrolyzing lipid peroxides [102]. PON transgenic cluster (PONs 1, 2, and 3) overexpression promotes plaque stability [3]. PON1^−/−^ mice show increased aortic O_2_^•−^ and leukocyte adhesion [77]; conversely, ApoE^−/−^/PON1^+/+^ mice show reduced atherosclerosis [78] (Table 2). These data support an anti-atherosclerotic role for PON1, probably by increasing the antioxidant activity of high-density lipoprotein (HDL) and reducing ox-LDL levels in the arterial wall [78]. The overexpression of PON1 in LDL^−/−^ adenovirus-mediated PON1 gene transfer mice reduced plaque volume [79] (Table 2). Human PON1 activity in serum is inversely related to the risk of ASCVD and stenosis requiring revascularization in patients with CAD [103,104]. The Met-Leu (M/L) 54 PON1 polymorphism is associated with reduced serum PON1 in DM subjects and correlates with increased ASCVD [87], the M/L55 and Gln-Arg (Q/R) 192 PON1 polymorphism is also associated with reduced serum PON1 activity and CAD [88,89] (Table 2). 

ApoE^−/−^/PON2^−/−^ mice show larger aortic atherosclerotic lesions and LDLs with higher lipid hydroperoxide content compared to ApoE^−/−^ mice [80], and PON2^−/−^ mice show high mitochondrial O_2_^•−^ levels in peritoneal macrophages and aortas [80] (Table 2). ApoE^−/−^ mice, injected with adenovirus-expressing human PON2, show significantly lower circulating lipid hydroperoxides. LDLs are less susceptible to oxidation, while HDL protect these from LDL oxidation [81] (Table 2). PON2 expression seems to be reduced in ECs and monocytes/macrophages from human carotid atherosclerotic lesions vs. healthy tissues [105].

Two mammalian ubiquitous Trx isoforms are known (Figure 2): Trx1 is a cytosolic and nuclear protein, whereas Trx2 is mitochondrial [106]. The Trx-related system reduces oxidized cysteine by interacting with the redox-active center of Trx (Cys-Gly-Pro-Cys), which, in turn, can be reduced by Trx reductase and NADPH [107] (Figure 2). EC-targeted Trx2^+/+^ mice show increased scavenging activity for H_2_O_2_ and O_2_^•−^ [72], ApoE^−/−^/Trx2^+/+^ mice show improved EC function and reduced atherosclerosis [72] and mice with targeted cardiac Trx2^−/−^ exhibit high oxidative status and vascular lesions [73,74] (Table 2). Trxs are expressed in human VSMCs of normal coronary arteries and are increased in atherosclerotic coronary arteries from autopsies, especially in macrophages [90] (Table 2), suggesting a possible role of Trx in the protection of human coronary arteries.

PRDX is a ubiquitous system of six mammalian isoforms in cytosol, mitochondria, and peroxisomes [65,108] (Figure 2). ApoE^−/−^/PRDX1^−/−^ and ApoE^−/−^/PRDX2^−/−^ mice display larger macrophage-rich aortic lesions [66] and accelerated plaque formation [67] (Table 2). ApoE^−/−^/PRDX4^+/+^ mice show reduced atherosclerotic lesions and ox-LDL levels [65] (Table 2). 

Three SOD isoforms are known: cytoplasmic SOD1, mitochondrial SOD2, and extracellular SOD3 [109,110] (Figure 2). These catalyze the dismutation of O_2_^•−^ into molecular O_2_ and H_2_O_2_ [111]. The role of SODs in atherosclerosis appears controversial [8]. In rabbit hearts perfused with high-dose SOD, the effects on atherogenesis seem to be dose-dependent [112] (Table 2). SOD1^−/−^ mice showed increased aortic O_2_^•−^ than WTs [75] (Table 2). ApoE^−/−^/SOD2^+/−^ mice showed increased atherosclerosis and plaque vulnerability [76] (Table 2). SOD3 expression in atherosclerotic vessels, VSMCs, and coronary ECs in humans is reduced in DM [113,114]. SOD3_R213G_ polymorphism is associated with reduced enzyme activity and increased ischemic heart disease [92]. A T-allele of rs2284659 variant in the promoter is associated with high SOD3 plasma levels and inversely correlates with MI incidence in type 1 (T1)DM and type 2 (T2)DM patients [93] (Table 2).

In conclusion, genetically modified mice overexpressing Cat, Cat^+^SOD1 [60], PRDX4 [60], or Trx2 in ECs [72] and the deletion of scavenger systems such as the nuclear factor [erythroid-derived 2]-like 2 related factors 2 (Nrf-2) [115], GPX1 [116], SOD2, and PRDX1 and 2 [3] indicate a protective role in atherosclerosis. The same patterns appear to be confirmed in fewer, descriptive human studies.

## 4. Human Circulating Biomarkers of Oxidative Stress

Several data arise from biomarker studies support a role for ROS in human ASCVD. Polyunsaturated lipids are susceptible to non-enzymatic oxidative damage, leading to F_2_-isoprostanes and malondialdehyde (MDA) [117,118] (Figure 1). The F_2_-isoprostane 8-epi-PGF_2α_ is non-enzymatically derived from ROS attack to the arachidonic acid of the cell’s membranes, and is stable and measurable in human urine [118]. It exerts pro-thrombotic and vascular-damaging actions by binding to the thromboxane (TX)A_2_ receptor (TP), which can then activate platelets and induce EC dysfunction and VSMC contraction [119,120,121] (Figure 3). A significant and strong association has consistently been reported between urinary 8-epi-PGF_2α_ excretion and serves as an in vivo biomarker of platelet activation, i.e., the urinary 11-dehydro-TXB_2_, a major enzymatic metabolite of platelet’s TXA_2_ [118,122], in human conditions of high CV risk, such as obesity [123,124], hypercholesterolemia [125], DM [126,127], pre-diabetes [128], essential thrombocythemia [129], hypertension [130], and cigarette-smoking (Table 3).

In addition, in 12,239 postmenopausal women followed over 18 years, urinary 8-epi-PGF_2α_ independently predicted CV mortality [133] (Table 3). 

MDA is a highly reactive dialdehyde generated from ROS-mediated lipid degradation (Figure 1) [157]. It can induce protein adducts and cross-linking [158], and is measurable in human blood [159]. Consistent with its lipid origin, plasma MDA and 8-epi-PGF_2α_ have been shown to be highly correlated in some studies (Table 3) [160]. MDA levels are increased in cigarette smoking [137,139], DM [134], CAD [135,138] patients, and they independently predicted MI and revascularization in CAD patients enrolled in the Prospective Randomized Evaluation of the Vascular Effects of Norvasc Trial [136] (Table 3).

Ox-LDLs are the end-product of non-enzymatic O_2_^•−^ modifications (Figure 1) to both LDL proteins and lipids and are measurable in human plasma [161,162]. Ox-LDLs contribute to foam cell development in the vessel wall and bind to macrophages via scavenger receptors [163] and to ECs through the lectin-like oxidized LDL receptor-1, increasing adhesion molecule binding [164] and platelet activation via the scavenger CD36 receptor [165] (Figure 3). Enhanced circulating ox-LDLs are reported in acute MI [140]. A meta-analysis of 8644 subjects with or without previous ASCVD showed that increased ox-LDLs are associated with ASCVD recurrence [144]; they also independently predicted carotid and femoral atherosclerosis and ASCVD in a prospective population-based survey of from 40- to 79-year-old men and women followed over 10 years [142]. They independently predicted CV death, MI, and angina in 238 CAD patients over 52 months [141], and predicted MI and CV death in acute coronary syndrome (ACS) patients [143] (Table 3).

Protein oxidation can be measured by nitrotyrosine derived from tyrosine nitration, ONOO^−^ and NO, in serum, plasma, and urine samples [166,167]. In a case-control study with 100 CAD patients, circulating nitrotyrosine levels were higher in CAD vs. non-CAD patients, and the rates of CAD and atherosclerosis were increased in the higher nitrotyrosine quartiles [146]. Nitrotyrosine is increased in T2DM patients as compared to healthy subjects [145] (Table 3). 

Protein carbonyls, the most frequent ROS-induced protein modification, are markers of the irreversible damage of lysine (Lys), arginine (Arg), proline (Pro), and threonine (Thr) residue oxidation [168], in a process named “primary protein carbonylation”. The end-product 2,4-dinitrophenylhydrazine [169,170] is stable and measurable in plasma [171]. Elevated circulating protein carbonyls were detected in T2DM [148,152], in hypercholesterolemia [151], and in CAD patients [149] (Table 3). Advanced glycation end products (AGEs) are protein carbonyls generated in the “secondary protein carbonylation” process through glycoxidation, and N^ε^-(carboxymethyl)lysine is the most abundant AGE [172], which is measurable in organic fluids and tissues [173]. AGEs cause cell damage by binding its receptor (RAGE), which activates nuclear factor-kappa B (NF-κB) [174], and seem to be involved in T2DM-related CV complications [147,150,153,155]. In a meta-analysis of seven prospective observational studies, including 3718 participants, increased circulating AGEs were associated with increased all-cause and CV mortality [156] (Table 3).

## 5. Pharmacological Interventions 

### 5.1. Antioxidant Compounds

**Figure 4:**
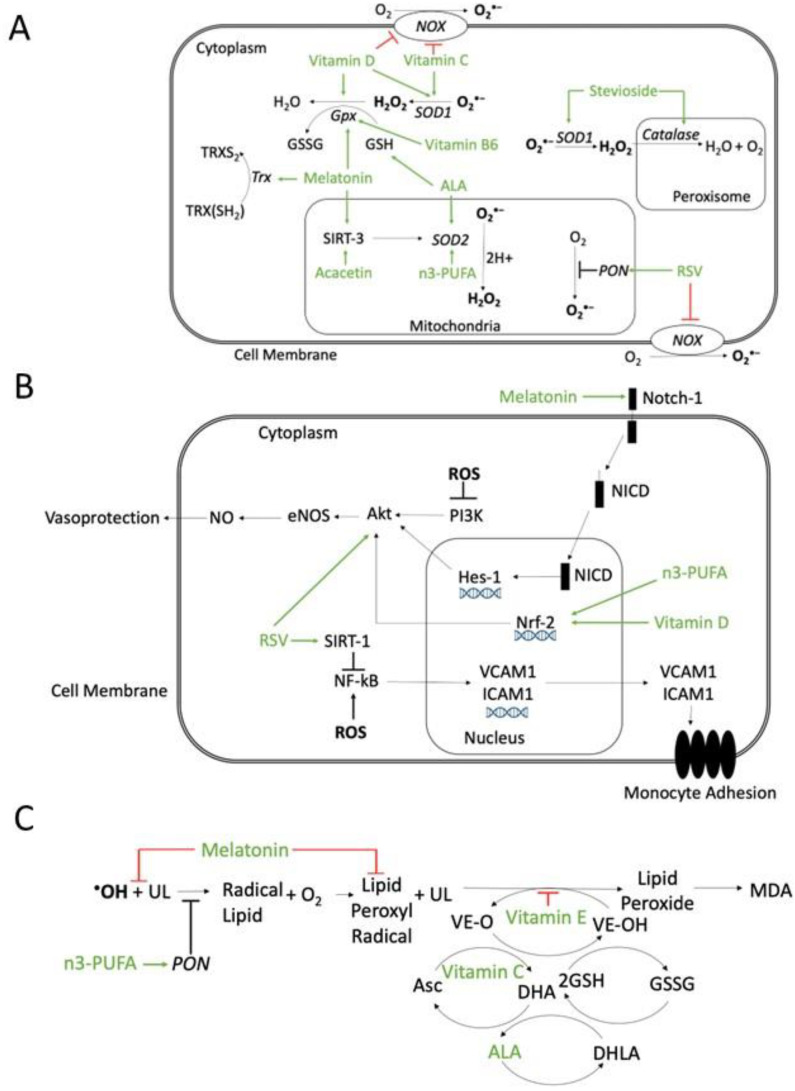
Possible mechanisms of action of antioxidant compounds. (**A**) Effects on ROS production or scavenger systems. Resveratrol (RSV) inhibits NADPH oxidase (NOX) and increases paraoxonase (PON) activity. Vitamin D and ascorbic acid (Vitamin C) inhibit NOX and increase superoxide dismutase (SOD) activity. Vitamin B6 is involved in glutathione peroxidase (GPX) synthesis. Alpha-lipoic acid (ALA) regenerates glutathione (GSH). Melatonin enhances glutathione peroxidase (GPX) and thioredoxin (Trx), reducing ROS. Melatonin and acacetin, through the mitochondrial Sirtuin-3 (SIRT-3) pathway, increase SOD scavenger activity. Stevioside enhances both SOD and peroxisomal catalase. n-3 polyunsaturated fatty acids (n3-PUFA) enhance SOD activity. (**B**) Inhibition of ROS-activated pathways involved in atherosclerosis. Melatonin can activate the Notch homolog 1 (Notch-1) pathway and restore, through hairy and enhancers of split-1 (HES-1), the Phosphatidyl Inositol 3-Kinase/Protein kinase B/Endothelial nitric oxide synthase (PI3K/Akt/eNOS) pathway, which is inhibited by ROS. ROS-induced PI3K/Akt/eNOS inhibition decreases NO and vasoprotection. Vitamin D and n3-PUFA, through the activation of nuclear factor erythroid 2-related factor 2 (Nrf-2), stimulates protein kinase B/Endothelial nitric oxide synthase/NO (Akt/eNOS /NO) pathway and NO release. Resveratrol (RSV) increases Akt activity, increasing NO and vasoprotection, and through Sirtuin-1(SIRT-1) pathway, inhibits ROS-induced nuclear factor kappa-light-chain-enhancer of activated B cells (NF-κB) deacetylation, which upregulates the nuclear transcription of vascular cell adhesion protein-1 (VCAM-1) and intercellular adhesion molecule-1 (ICAM-1), leading to monocyte adhesion. (**C**) Inhibition of lipid peroxidation. Melatonin can scavenge ^•^OH and lipid peroxyl radicals. Vitamin E can scavenger lipid peroxyl radicals. Vitamin C can regenerate preferentially the Vitamin E radical and the ascorbic acid radical can be regenerated by GSH. ALA can regenerate both ascorbate and tocopherol radicals. n3-PUFA increases PON activity and reduces lipid peroxidation. Abbreviations: Asc: ascorbic acid (reduced); DHA: dehydroascorbic acid (oxidized); DHLA: hydrolipidic acid; GSSG: oxidized glutathione; MDA: malondialdehyde; NICD: notch 1 intracellular domain; UL: unsaturated lipid; VE-O: vitamin E oxidized; VE-OH: vitamin E reduced. Green arrows: activation; red block signs: inhibition.

Several molecules with antioxidant properties have been studied in animal models of atherosclerosis and in humans (Figure 4). 

Melatonin appears to increase the activity of antioxidant enzymes such as SOD and GPX, through Sirtuin (SIRT)-3 [175]. Resveratrol is a phytoalexin derived from grapes [176], likely acting via several mechanisms: the downregulation of NOX expression and activity, mitochondrial O_2_^•−^ reduction [177,178], and increased PON1 activity (Figure 4A). Vitamin E refers to a group of 8 different compounds, 4 tocopherols, and 4 tocotrienols, exerting their antioxidant action by scavenging lipid peroxyl radicals through hydrogen donation from the phenolic group of the chromanol ring (Figure 4C). Vitamin E inhibits peroxyl radicals before they react with lipids such as cholesterol, cholesterol esters, fatty acids, and phospholipids [179]. Different Vitamin E forms, with the un-substituted 5-position or with the methyl-group in five positions, can also trap reactive NO species [180,181]. Vitamin D inhibits NOX, upregulates several scavenging systems, such as SOD, GPX, and Cat [182] (Figure 4A), increases NO and the activation of phosphoinositide 3-kinases/protein kinase B (PI3K/Akt) [183] (Figure 4B). Ascorbic acid, i.e., Vitamin C, appears to exert diverse anti-oxidant effects [184] through the inhibition of NOX and XO, SOD activation [185]. Ascorbic acid can preferentially regenerate the Vitamin E radical, while the ascorbic acid radical can be regenerated by GSH [186,187] (Figure 4C). Vitamin B6 is water-soluble; its active form is a cofactor [188], which catalyzes homocysteine trans-sulphuration, contributing to the homocysteine production required for GSH synthesis [189], and is involved in GPX synthesis [190] (Figure 4A). Alpha-lipoic acid (ALA) and its reduced form can regenerate anti-oxidant molecules such as GSH, Vitamin C, Vitamin E, and cofactor Q10 (CoQ10) [191] (Figure 4). Stevioside, a common sweetener [192], contains polyphenol, can increase intracellular reduced GSH, upregulates SOD and Cat and decreases lipid peroxidation [193] (Figure 4A). Acacetin is a natural flavone of plant pigments [194] and can increase SOD2 [195], and Trx activity [196] (Figure 4A). N-3 polyunsaturated fatty acids (n3-PUFAs), such as eicosapentaenoic acid (EPA) and docosahexaenoic acid (DHA), seem to have different effects: in mitochondria, DHA reduces the cytochrome complex IV activity and increases SOD [197]. PUFAs upregulate the Nrf-2 transcription that leads to antioxidant gene expression [198] and enhances NO synthesis in ECs [199] (Figure 4A). 

Some dietary habits appear to be associated with antioxidant properties such as fish consumption, which is likely related to high PUFAs content [200], and some fish proteins also have a scavenger effect by inhibiting lipid peroxidation [201] (Figure 4C). The Mediterranean diet is rich in green vegetables, fish, and fruit, containing polyphenolic compounds, and PUFAs [202], including nuts and virgin olive oil, which can increase PON-1 activity, reducing lipid peroxidation [203] (Figure 4C).

### 5.2. Studies in Animals

In streptozotocin (STZ)-treated rats that develop DM, the supplementation of melatonin (20 mg/kg once daily (od) per os) for 8 weeks could recover Notch homolog-1 translocation associated/hairy and enhancers of split/protein kinase B (Notch1/Hes/Akt) signal in an I/R injury model and enhanced SOD in aortic VSMC [204] (Figure 4B). In the same animal model, intraperitoneal melatonin (10 mg/kg od) increased SOD and decreased MDA in erythrocytes [205,206] (Figure 4A). 

In DM-induced KsJ-*db/db* mice, resveratrol added to the chow (0.3% *w*/*w*) reduced adhesive molecule expression in aortic ECs [207]. In STZ-DM LDLr^−/−^ mice, resveratrol added to HFD (0.2% *w*/*w*) decreased monocyte MCP-1-dependent activation in the aortic root [208]. In ApoE^−/−^ mice, resveratrol (10 mg/kg od) for 6 weeks decreased macrophage differentiation, increased monocytes GSH and decreased atherosclerosis [209]. In C57BL/6 mice on HFD, resveratrol (10 mg/kg in the chow) could restore the integrity of aortic media and recover EC function through the phosphorylation of the Akt/eNOS pathway [210].

Vitamin E (100 mg/od) halved the mortality of HFD-fed mice, and decreased macrophages in atherosclerotic lesions and circulating MDA [211].

In ApoE^−/−^ STZ-induced-DM mice, ALA (1.65 g/kg od) reduced plasma lipid peroxidation, and increased erythrocyte GSH, and PON activity, slowing atherosclerosis [212]. 

In obese mice, stevioside (10 mg/kg od) for 12 weeks improved glucose transport and reduced autoantibodies against MDA-modified LDL [193], decreased ox-LDL in obese insulin-resistant LDLr^−/−^ mouse plaques [213].

In ApoE^−/−^ STZ-DM mice, acacetin (20 mg/kg twice daily) increased SOD and attenuated atherosclerotic lesions [214].

Compared to ApoE^−/−^ mice fed with corn oil, ApoE^−/−^ mice fed with fish oil containing n-3 PUFA (32.5 g/100 g total fatty acids) and n-6 PUFA (9.6 g/100 g total fatty acids) reduced atherosclerotic lesions, increased liver GSH and Cat levels [215] and lowered P-selectin and VCAM-1 expression in aorta [216]. Moreover, ApoE^−/−^ mice fed with n3-PUFA-enriched diet had a higher expression of eNOS and reduced O_2_^•−^ in the aorta versus a corn-oil-enriched diet [217]. The supplementation of a western diet with 5% EPA to LDLr^−/−^ mice was associated with lower macrophages’ infiltration in the aorta [218]. In HFD-fed ApoE^−/−^ mice, the antioxidant mitoquinone, a ubiquinone analogue, reduced DNA damage and atherosclerotic lesions [219].

### 5.3. Intervention Studies in Humans

Several studies in humans investigated a possible benefit of antioxidants by using biomarkers known as surrogates of either CV protection or CV events, which are summarized in Table 4 and Table 5, respectively.

In a small, double-blind, placebo-controlled, randomized clinical trial (RCT) in 60 DM subjects with CAD, supplementation with melatonin (10 mg od) for 12 weeks increased plasma GSH, NO, and decreased MDA and C-reactive protein (CRP) vs. placebo [220] (Table 4).

**Table 4:** Randomized clinical trials and meta-analyses of antioxidant compounds and dietary intervention on cardiovascular functional surrogates or oxidative-stress biomarkers.

Study (Year)	Study Population	Design and Study Duration	CV Functional Surrogates or Oxidative Stress Biomarkers	Results
Ashor et al. (2014) [221]	Adults with T1DM and T2DM, hypertension, heart failure and healthy subjects (*n* = 1129)	Meta-analysis of 44 RCT on vitamin C (<500 mg/od to >2 g/od) on endothelial function. Treatment duration: 1 day to 8 weeks	Endothelial function evaluated as FMD, plethysmography, pulse wave analysis and forearm blood flow	Standardized mean difference for endothelial function: 0.50, 95% CI = 0.34–0.66; *p* < 0.001
Montero et al. (2014) [222]	T2DM (*n* = 296)	Meta-analysis of 10 trials: Vitamin E or Vitamin C (*n* = 148) vs. placebo (*n* = 148) Treatment duration: 2–52 weeks	Endothelial function, evaluated as FMD or PORH or plethysmography	Standardized mean difference for endothelial function: 0.35, 95% CI = −0.17–0.88; *p* = 0.18
Derosa G et al. (2016) [223]	T2DM (*n* = 105)	Randomized study: alpha lipoic acid (ALA) 600 mg/od (*n* = 54) Vs. placebo (*n* = 51) Follow-up: 3 months	Serum SOD, erythrocyte GPX, plasma MDA	SOD comparison of within-group variations: ALA 16.7 U/mL vs. placebo 1.9 U/mL; *p* < 0.05 GPX comparison of within-group variations: ALA 22.4 EE/U vs. placebo 0.7 EE/U; *p* < 0.05 MDA comparison of within-group variations: ALA −8.9 nmol/mL vs. placebo −3.1 nmol/mL; *p* < 0.05
Imamura et al. (2017) [224]	T2DM (*n* = 50)	Randomied study: Resveratrol 100 mg/od (*n* = 25) vs. placebo (*n* = 25) Treatment duration: 12 weeks	Arterial stiffness assessed by cardio-ankle vascular index	Within-group difference in cardio-ankle vascular index: resveratrol −0.4 ± 0.7 vs. placebo 0.1 ± 0.5; *p* < 0.01
Mansournia et al. (2018) [225]	T2DM (*n* = 1053)	Meta-analysis of 33 studies: vitamin D vs. placebo Follow-up: 6 weeks–12 months	Serum CRP, eNOS, MDA	CRP-weighted mean difference between vitamin D vs. placebo: −0.27, 95% CI = −0.35–0.20; *p* < 0.001 NO-weighted mean difference between between vitamin D vs. placebo: 4.33, 95% CI = 0.96–7.70; *p* < 0.001 MDA-weighted mean difference between between vitamin D and placebo: –0.43, 95% CI = −0.62–0.25; *p* < 0.001
Sattarinezhad et al. (2018) [226]	T2DM and nephropathy (*n* = 60)	Randomized study: Resveratrol 500 mg/od (*n* = 30) vs. placebo (*n* = 30) Follow-up: 90 days	Serum markers of NO, mSOD and MDA	NO markers’ comparison of within-group variation: resveratrol 4.4 ± 5.61 μmol/l vs. placebo −0.5 ± 5.0 μmol/L; *p* < 0.01 SOD comparison of within-group variation: resveratrol 4.8 ± 5.3 U/L vs. placebo −4.2 ± 9.3 U/L; *p* < 0.01 MDA comparison of within-group variations: resveratrol −0.4 ± 0.9 nmol/mL vs. placebo 0.9 ± 1.3 nmol/mL; *p* < 0.01
Seyyedebrahimi et al. (2018) [227]	T2DM (*n* = 60)	Randomized study: Resveratrol 800 mg/od (*n* = 30) vs. placebo (*n* = 30) Follow-up: 2 months	Ferric-reducing ability in plasma (FRAP)	Percentage of FRAP change: resveratrol 44.41 ± 138.52% vs. placebo 15.30 ± 88.72%; *p* = 0.002
Hoseini et al. (2019) [228]	T2DM (*n* = 46)	Randomized study: Resveratrol 500 mg/od (*n* = 23) vs. placebo (*n* = 23) Follow-up: 4 weeks	Plasma MDA and ferric-reducing ability (FRAP)	Difference between resveratrol and placebo MDA: −0.21 μmol/L, 95% CI = −0.41–0.005; *p* = 0.04 FRAP: 58.88 mmol/L, 95% CI = 17.33–100.44; *p* = 0.006
Mendoza-Nùñez et al. (2019) [229]	Adults aged 60–74 years with T2DM (*n* = 135)	ALA 600 mg/od (*n* = 50) vs. placebo (*n* = 50) Follow-up: 6 months	Erythrocyte SOD/GPx, plasma 8-epi-PGF_2α_	Comparison of within-group variations SOD/GPx: ALA −0.004 vs. placebo −0.005 vs. control 0.005; *p* < 0.05 Comparison of within-group variations 8-epi-PGF_2α_: ALA −43 vs. placebo −29 vs. control 13; *p* < 0.05
Raygan et al. (2019) [220]	T2DM with BMI ≥ 25 g/m^2^ and coronary heart disease, with 2- and 3- vessels (*n* = 60)	Randomized study: Melatonin 10 mg/od (*n* = 30) vs. placebo (*n* = 30) Follow-up:12 weeks	Plasma GSH, NO and MDA	Within-group change of GSH Melatonin +64.7 ± 105.7 mmol/L Placebo −11.1 ± 137.6 mmol/L; *p* = 0.02 Comparison of within-group variations NO melatonin +0.9 ± 4.7 mmol/L vs. placebo −3.3 ± 9.6 mmol/L; *p* = 0.03 Comparison of within-group variations MDA melatonin −0.2 ± 0.3 mmol/L vs. placebo +0.1 ± 0.5 mmol/L; *p* = 0.007
Dalan et al. (2020) [230]	T2DM (*n* = 166)	Randomized study: Vitamin E 400 UI/od (*n* = 84) vs. placebo (*n* = 82) Follow-up: 24 weeks	Endothelial function assessed as peripheral arterial tonometry- reactive hyperaemia index (EndoPAT-RHI)	Difference of EndoPAT-RHI Vitamin E vs. placebo −0.02, 95% CI −0.10–0.06; *p* = 0.690

Abbreviations: ALA: alpha-lipoic acid; BMI: body mass index; CI: confidence interval; CRP: C-reactive protein; CV: cardiovascular; 8-epi-PGF_2α_:8-epi-prostaglandin F_2α_; eNOS: endothelial nitric oxide synthase; FRAP: ferric-reducing ability; FMD: flow-mediated dilation; GPX: glutathione peroxidase; GSH: glutathione; HDL: high-density lipoprotein; MDA: malondialdehyde; od: once daily; PORH: post-occlusive reactive hyperaemia; RCT: randomized clinical trial; SOD: superoxide dismutase; T1DM: type 1 diabetes mellitus; T2DM: type 2 diabetes mellitus.

In another small study, patients with DM and CAD that were randomized to resveratrol (500 mg/od, *n* = 23) for 4 weeks showed an increased total antioxidant capacity in plasmas, as assessed by ferric-reducing ability (FRAP) and reduced MDA versus controls [228](Table 4). Two-month resveratrol (800 mg/od) increased FRAP in 48 DM subjects [227]; higher peripheral eNOS and GPX levels were reported in 60 DM subjects with nephropathy taking resveratrol (500 mg/od for 3 months) vs. placebo [226] (Table 4). Furthermore, resveratrol supplementation (100 mg/od for 12 weeks) was associated with a change in the cardio-ankle vascular index [231] in 50 subjects with T2DM vs. placebo [224] (Table 4). Moreover, in 135 T2DM patients, ALA (600 mg/od for 6 months) consistently increased erythrocyte SOD and GPX activity vs. placebo [229]; in another study on 105 T2DM subjects ALA (600 mg/od for 3 months) improved metabolic control, increased serum SOD and erythrocyte GPX activity and decreased plasma MDA [223] (Table 4).

Vitamin E (400 UI/od for 24 weeks) supplementation in 187 T2DM subjects did not modify vascular motility or ROS generation [230] (Table 4). A meta-analysis on supplementation with either Vitamin C or E in 296 subjects with T2DM did not show any difference in EC-dependent vasodilation as compared to placebo [222] (Table 4). However, the supplementation of Vitamin E 100 or 600 mg/od for 14 days in 22 hypercholesteremic patients was associated with a dose-dependent, significant decrease in urinary 8-epi-PGF_2α_ [125]. A systematic review and meta-analysis of 1129 subjects showed a positive effect of Vitamin C on EC-dependent flow-mediated dilation, forearm blood flow, and pulse wave analysis (Table 4) [221]. Notably, the positive effect of Vitamin C was observed in healthy subjects, in whom EC dysfunction was induced by glucose, methionine and endotoxins, and a very high dose of Vitamin C (2600 mg) was used [221]. A meta-analysis of the effect of 33 placebo-controlled RCTs on 1053 DM participants showed that Vitamin D supplementation (between 200 UI/od to 50,000 UI/monthly), was associated with decreased serum CRP and MDA, and increased circulating markers of NO and GSH [225] (Table 4).

While some studies using biomarkers or indirect indexes of CV diseases showed some effect of the antioxidant compounds, RCTs with hard endpoints were largely negative. The Women’s Health Study randomized 39,000 healthy women taking Vitamin E (600 UI every other day (eod)) or placebo and failed to show any reduction in MI, stroke or CV death over a mean of 10.1 years [232] (Table 5). 

**Table 5:** Randomized clinical trials and meta-analyses of antioxidant compounds and dietary intervention on cardiovascular outcomes.

Study (Year)	Study Population	Design and Study Duration	Primary Endpoints	Results
De Lorgeril et al. (1994) [233]	Adults aged < 70 yrs with a MI within 6 months (*n* = 605)	Randomized study: Mediterranean alpha-linolenic acid-rich diet (*n* = 302) versus Usual diet (*n* = 303) Mean follow-up: 27 months	Non-fatal acute MI and CV death	Primary Endpoint Mediterranean diet *n* = 8 Usual diet *n* = 33 RR 0.27, 95% CI 0.12–0.59, *p* = 0.001
Yusuf et al. (2000) [234]	High CV Risk for previous CV events or T2DM+1 CV risk factor (*n* = 9541)	Randomized study: Vitamin E 400 UI/od (*n* = 4761) vs. placebo (*n* = 4780) Mean follow-up: 4.5 years	MI, stroke, or CV death	Primary endpoint: Vitamin E *n* = 772 (16.2%) Placebo *n* = 739 (15.5%) RR: 1.05, 95% CI 0.95–1.16; *p* = 0.33
Knoops et al. (2004) [235]	Healthy elderly from 2 European cohorts (FINE *n* = 726 and SENECA *n* = 1613)	Pooled analysis on the effect of Mediterranean diet, quitting smoking and engaging physical activity on mortality Mean follow-up: 10 years	All-cause mortality, Death from CAD, CV death	All-cause mortality Mediterranean diet HR: 0.77, 95% CI 0.68–0.88 Death from CAD Mediterranean diet HR: 0.61, 95% CI 0.43–0.88 CV Death Mediterranean Diet HR: 0.71, 95% CI 0.58–0.88
Whelthon et al. (2004) [236]	Adults with and without CV disease (*n* = 228,864)	Metanalysis of 19 observational studies (14 cohort studies and 5 case-control studies) comparing regular fish consumption (mean intake 36 g/od or 2.2 servings/week) vs. little/no fish consumption Mean follow-up of cohort studies: 15 years	Fatal and Total CAD	Fatal CAD Regular Fish consumption RR: 0.83, 95% CI 0.76 to 0.90; *p* < 0.005 Total CAD Regular Fish Consumption RR: 0.86, 95% CI 0.81–0.92; *p* < 0.005
Lee et al. (2005) [232]	Healthy women aged ≥ 45 (*n* = 39,876)	Randomized study: Vitamin E 600 UI/eod (*n* = 19,937) vs. placebo (*n* = 19,939) Mean follow-up: 10.1 years	Nonfatal MI, nonfatal stroke, or CV death	Primary endpoint: Vitamin E *n* = 482 (2.4%) Placebo *n* = 517 (2.5%) RR: 0.93, 95% CI 0.82–1.05; *p* = 0.26
Cook et al. (2007) [237]	Female aged ≥ 40 with previous CV event or with ≥3 CV risk factors (hypertension, high cholesterol, DM, history of MI, BMI ≥30 kg/m^2^, current cigarette smoking) (*n* = 8171)	Randomized study, 2X2 Factorial design: Vitamin E 600 UI/eod (*n* = 4087), Vitamin C 500 mg/od (*n* = 4083) vs. placebo (*n* = 4084) Mean follow-up: 9.4 years	MI, stroke, CABG or PTCA, CV death	Primary endpoint: Vitamin E *n* = 708 (17.3%) Placebo *n* = 742 (18.1%) RR: 0.94, 95% CI 0.85–1.04; *p* = 0.23 Vitamine C *n* = 731 (17.9%), Placebo *n* = 719 (17.5%), RR: 1.02, 95% CI 0.92–1.13; *p* = 0.71
Sesso et al. (2008) [238]	Male aged ≥ 50 years, including 5.1% with prevalent CV disease, as MI and stroke (*n* = 14,641)	Randomized study, 2 × 2 factorial Design: Vitamin E 400 UI/eod (*n* = 7329) + Vitamin C 500 mg/od (*n* = 7315) vs. placebo (*n* = 7312 vs. Vitamin E or *n* = 7326 vs. Vitamin C) alone Mean follow-up: 8.0 years	Non-fatal MI, non-fatal stroke, CV death	Primary endpoint: Vitamin E *n* = 620, 1.09 events per 1000 person–years Placebo *n* = 625, 1.09 events per 1000 person–year HR: 1.01, 95% CI 0.90–1.13; *p* = 0.86 Vitamin C *n* = 619, 1.08 events per 1000 person–years Placebo *n* = 626, 1.09 events per 1000 person–years HR: 0.99, 95% CI 0.89–1.11; *p* = 0.91
Myung et al. (2013) [239]	Adults with and without CV disease (*n* = 294,478)	Metanalysis of 50 RCT evaluating the effect of several compounds (Vitamins Q10 coenzyme, calcium, n3-fatty acids) Follow-up: 6 months–12 years	CV death, MI, stroke, angina, sudden cardiac death	Primary endpoint All compounds RR 1.00, 95% CI 0.98–1.02 Vitamin B6 RR 0.92, 95% CI 0.85–0.99
Bowman et al. (2018) [240]	T2DM without ASCVD (*n* = 15,480)	Randomized study: n-3 fatty acid 1 g/od (*n* = 7740) vs. placebo (*n* = 7740) Mean follow-up: 7.4 years	Non-fatal MI or stroke, TIA, vascular death	Primary endpoint n-3 fatty acid group *n* = 689 (8.9%) Placebo *n* = 712 (9.2%) RR: 0.97, 95% CI 0.87–1.08; *p* = 0.55
Estruch et al. (2018) [241]	Subjects at high CV risk (T2DM or ≥3 CV risk factors, as smoking, hypertension, elevated LDL cholesterol, low HDL cholesterol, overweight or obesity, or a family history of premature CHD) (*n* = 7447)	Randomized study: mediterranean diet with extra-virgin olive oil integration (*n* = 2543) vs. mediterranean diet with mixed nuts integration (*n* = 2454) vs. dietary fat reduction advice as control (*n* = 2450) Median follow-up: 4.8 years	MI, stroke, CV death	Primary endpoint Mediterranean diet with extra-virgin olive oil *n* = 98 (3.8%) Incidence rate 8.1 per 1000 person–years HR vs. control: 0.69, 95% CI 0.53–0.92; *p* < 0.05 Mediterranean diet with nuts *n* = 83 (3.4%) Incidence rate 8.0 per 1000 person–years HR vs. control: 0.72, 95% CI 0.53–0.94; *p* < 0.05 Control group *n* = 109 (4.4%) Incidence 11.2 per 1000 person–years
Manson et al. (2019) [242]	Men aged ≥50 years and women aged ≥ 55 years without CV disease (*n* = 25,871)	Randomized study: Vitamin D 2000 UI/od + n-3 fatty acid 1 g/od (*n* = 12,927) vs. placebo (*n* = 12,944) Median follow-up: 5.3 years	MI, stroke, CV death	Primary endpoint Vitamin D + n-3 fatty acid group *n* = 96 (0.03%) Placebo group *n* = 409 (0.03%) HR: 0.97, 95% CI 0.85–1.12; *p* = 0.69
Khan et al. (2021) [243]	Adults with and without CV disease (*n* = 149,051)	Metanalysis of 38 RCTs evaluating the effect of EPA alone (4 RCTs) or of EPA+DHA (34 RCTs) vs. placebo or low-dose fatty acid supplementation. Mean follow-up: 2.0 years	CV death, non-fatal MI, CHD	CV death Overall RR 0.93, lower limit 0.88-upper limit 0.98; *p* = 0.01 EPA RR 0.82, lower limit 0.68, upper limit 0.99; *p* = 0.04 EPA+DHA RR 0.94, lower limit 0.89, upper limit 0.99; *p* = 0.02 Non-fatal MI Overall RR 0.87, lower limit 0.81, upper limit 0.93; *p* < 0.01 EPA RR 0.72, lower limit 0.62, upper limit 0.84; *p* < 0.01 EPA+DHA RR 0.92, lower limit 0.85, upper limit 1.00; *p* = 0.05 CHD Overall RR 0.91, lower limit 0.87, upper limit 0.96; *p* < 0.01 EPA RR 0.73, lower limit 0.62, upper limit 0.85; *p* < 0.01 EPA+DHA RR 0.94, lower limit 0.89, upper limit 0.99; *p* = 0.01
Mohan et al. (2021) [244]	Adults with and without CV event (PURE *n* = 147,645 ONTARGET/TRASCEND *n* = 31,491 ORIGIN *n* = 12,422)	Pooled analysis of individual participant data from a cohort study and 3 RCTs (ONTARGET, TRASCEND, ORIGIN) comparing high fish intake (≥175 g/weekly) vs. little/no fish intake (<50 g/monthly) Median follow-up: PURE: 9.1 years; ONTARGET/TRASCEND: 4.5 years; ORIGIN 6.2 years	MI, stroke, congestive heart failure, or sudden death, all-cause mortality	Primary Endpoints PURE Subjects without prior CV event >175 g/weekly fish HR: 0.94, 95% CI 0.88–1.01 Subjects with prior CV event >175 g/weekly fish HR: 0.89, 95% CI 0.74–1.06 ONTARGET/TRASCEND Subjects with prior CV event >175 g/weekly fish HR: 0.88, 95% CI 0.80–0.97; *p* < 0.05 ORIGIN Subjects without prior CV event >175 g/weekly fish HR: 0.94, 95% CI 0.88–1.04 Subjects with prior CV event >175 g/weekly fish HR: 0.86, 95% CI 0.80–0.92; *p* < 0.05

Abbreviations: BMI: body mass index; CABG: coronary artery bypass grafting; CAD: coronary artery disease; CI: confidence interval; CV: cardiovascular; eod: every other day; HDL: high-density lipoprotein; HR: hazard ratio; LDL: low-density lipoprotein; MI: myocardial infarction; od: once daily; PTCA: percutaneous transluminal coronary angioplasty; RCT: randomized clinical trial; RR: relative risk; T2DM: type 2 diabetes mellitus; TIA: transient ischemic attack.

Similarly in the Heart Outcomes Prevention Evaluation (HOPE) RCT, Vitamin E (400 UI/ od) did not reduce MI, stroke, and CV death in 9541 subjects with a previous CV event or DM over 4.5 years [234] (Table 5). The Physicians’ Health Study II RCT studied a combination of Vitamin E (400 IU/eod) and C (500 mg/od) on MI, stroke, and CV death in 14,641 healthy US male physicians over 8 years, but no benefit was observed versus placebo [238] (Table 5). The Women’s Antioxidant Cardiovascular Study tested Vitamin E (600 IU/od), C (500 mg/od), and beta-carotene (50 mg/eod) on the prevention of MI, stroke, coronary revascularization, or CV death in 8171 women with a history of ASCVD or at least three CV risk factors and failed to show any benefit [237] (Table 5).

A meta-analysis of RCT on the supplementations on Vitamin A, E, C, beta-carotene, and selenium suggested that the some compounds could even increase all-cause mortality, while selenium and ascorbic acid had no effect [245]. The Vitamin D and omega-3 Trial investigated vitamin D cholecalciferol (2000 IU/od) and n-3 FA (1 g/od) on the prevention of MI, stroke, or CV death versus placebo over 5.3 years, showing no benefit [242] (Table 5).

A Study of Cardiovascular Events in Diabetes (ASCEND) RCT randomized n-3 fatty acid (1 g/od) vs. placebo, in >15,000 DM subjects with no evidence of symptomatic CV diseases, and there was no CV benefit associated with omega-3 over 7.4 years [240] (Table 5). Recently, a meta-analysis including 38 RCTs demonstrated that supplementation with EPA (from 1.8 to 4.0 g/od), or with a combination of EPA and DHA (0.4 to 5.5 g/od), was associated with a reduction in CV mortality, non-fatal MI, and CHD, with a higher reduction observed with EPA monotherapy [243]. However, results regarding the effect of EPA and DHA combination were not confirmed by the same authors when older trials with suboptimal statin therapy were excluded from the analysis: EPA plus DHA was, in fact, not associated with reduced CV death or non-fatal CV events [243]. 

In a large meta-analysis, including 50 studies and 294,478 participants, the supplementation of diverse antioxidants, including CoQ10, calcium, zinc, and n-3 fatty acids, did not reduce major CV events vs. no treatment or placebo in both primary and secondary CV prevention. Even in subgroup analyses of the type of intervention, outcome, quality of antioxidant, duration of treatment, and combined vs. single Vitamin administration, no CV benefit was detected, except a slight CV reduction for low-dose Vitamin B6 (RR 0.92, 95% CI from 0.85 to 0.99) [239] (Table 5).

Despite the largely negative RCT data, the Mediterranean diet and fish consumption, known for their antioxidant properties [246], have been associated with a lower risk of CV events or death in large epidemiological studies. Healthy Ageing, a longitudinal study in Europe, including 2239 healthy elderly subjects from two large surveys, followed for a mean of 10 years, showed that the Mediterranean diet was associated with significantly lower risk of all-cause mortality and CV diseases [235] (Table 5). In the Prevención con Dieta Mediterránea (PREDIMED) Study, 7447 subjects at high CV risk but without CV event were assigned to a Mediterranean diet with extra-virgin olive oil integration, a Mediterranean diet with mixed-nuts integration or a dietary fat reduction as control (Table 5). The primary endpoint of major CV events (MI, stroke, or CV death) was reduced (HR 0.69, 95% CI 0.53–0.92) for the Mediterranean diet with extra-virgin olive oil and for a Mediterranean diet with nuts (HR 0.72; 95% CI: 0.53–0.94) versus the control diet [241]. In the Lyon Diet Heart Study, a secondary prevention trial including 605 subjects with a recent MI, after a mean of 27 months, found that a Mediterranean diet was associated with significantly lower CV death and acute MI [233] (Table 5).

In a meta-analysis, including observational data, comparing regular fish consumption vs. little or no fish intake, fish consumption was associated with a relative risk of 0.83 (95% CI 0.76–0.90) for fatal CAD, and of 0.86 (95% CI 0.81–0.92) for total CHD [247] (Table 5). Recently, a meta-analysis including data from a large-scale cohort study and three RCTs showed that fish intake (at least 175 g/week) was associated with lower major CV disease, CV, non-CV and total mortality as compared with ≤50 g/month intake [248] (Table 5). 

## 6. Conclusions

Animal studies strongly support a causal link between some enzymes and systems of generation and/or the clearance of ROS with atherosclerosis development, supporting the notion that controlling ROS is an appropriate goal for therapeutic interventions to prevent ASCVD. However, studies on antioxidant substances in humans led to inconsistent evidence regarding the effect on reducing and preventing ASCVD development or complications to date, while some studies using functional tests or soluble biomarkers have shown a positive impact on the same compounds.

Negative RCTs have helped to identify the pitfalls of the current approaches and how to design future interventions. Problems associated with RCTs can be agent concentrations, exposure time, and ASCVD status (early vs. late), while ROS are not always damaging to cell function since they can also regulate cell homeostasis, and their role is very much cell- and tissue-dependent. In addition, different ROS may have different roles (H_2_O_2_ vs. O_2_^•−^). GKT137831 (setanaxib), a promising NOX1/4 inhibitor, is currently in phase II clinical trials for DM kidney disease [249]. 

In conclusion, while animal models have identified several targets along the paths of ROS production and clearance, intervention RCTs are still lacking, while the dietary habits associated with a possible reduction in ROS tone have shown CV benefits. Future research will have to unravel these gaps, and find the reasons for, and the way to overcome, these inconsistent results.

## Figures and Tables

**Table 1:** ROS production and atherosclerosis in animal models and in human diseases.

Genetic Background and/or Experimental Setting	Phenotype
Animal Models	
ApoE^−/−^	↑ NOXA-1, NOX2, and O_2_^•−^ in the aortic atherosclerotic lesions, as assessed by DHE and L-012 vs. WT mice [10,11]
ApoE^−/−^/NOX1^−/−^	↓ O_2_^•−^ levels in the aorta, as assessed by L-012, macrophage infiltration and MDA in atherosclerotic lesions vs. ApoE^−/−^ [12,13]
ApoE^−/−^ on HFD and a NOX2 inhibitor	↓ O_2_^•−^ levels assessed by DHE and atherosclerotic lesion areas vs. ApoE^−/−^ [14]
NOX2^−/−^ with vascular wire-injury	↓ O_2_^•−^ from platelets and in the aorta (by DCF and DHE, respectively), ↓ macrophage infiltration, cellular proliferation, and platelet adhesion on injured aortas vs. WT [15,16]
ApoE^−/−^/NOX2^−/−^	↓ O_2_^•−^ as assessed by L-012, macrophage infiltration and number of lesions in the aorta ↑ NO in the aorta vs. ApoE^−/−^ [11]
ApoE^−/−^/EC NOX2^+/+^	↑ O_2_^•−^ levels, as assessed by L-012 and DHE, VCAM-1, and macrophage infiltration into early aortic lesions vs. ApoE^−/−^ [17]
ApoE^−/−^/p47*phox*^−/−^	↓ O_2_^•−^ levels, as assessed by DHE, macrophage infiltration, and atherosclerotic lesion burden vs. ApoE^−/−^ [18]
ApoE^−/−^/gp91*phox*^−/−^	↓ O_2_^•−^ levels, as assessed by DHE and atherosclerosis ↑ NO in the aorta vs. ApoE^−/−^ [11]
NOX1^−/−^/NOX2^−/−^/NOX4^−/−^	↓ O_2_^•−^ from platelets, as assessed by EPR, platelet adhesion and aggregation in vitro vs. WT platelets [19]
ApoE^−/−^/LDLr^−/−^	↑ NOX4 and O_2_^•−^ in the aortic lesions vs. WT [20]
Rabbits on HFD with or withour XO inhibitor	↓ O_2_^•−^ levels in the aorta, assessed by L-012 ↑ endothelium-dependent relaxation in response to acetylcholine vs. HFD animals [21]
ApoE^−/−^ on a XO inhibitor	↓ O_2_^•−^ as assessed by DHE, chemokine CK, IL-1α, IL-1β, and MCP-1 expression, and atherosclerotic lesions vs. ApoE^−/−^ [22,23]
ApoE^−/−^/MPO^−/−^ bone marrow	↓ O_2_^•−^ as assessed by DHE and atherosclerotic lesions, ↑ NO in the aorta vs. ApoE^−/−^ [24]
LDLr^−/−^ transplanted with MPO^−/−^ bone marrow	↑ Macrophage infiltration and atherosclerotic lesion area vs. LDLr^−/−^/MPO WT [25]
MicroRNA-210^−/−^	↑ Mitochondrial ROS after I/R vs. WT [26]
**Human studies**	
NOX mRNA expression	↑ NOX2 and NOX4 in coronary arteries from CAD patients vs. non-CAD [27,28]
Congenital NOX2 deficiency	↓ Atherosclerosis, ox-LDL, and 8-epi-PGF_2α_ vs. controls [29] ↓ O_2_^•−^ as assessed by L-012 and 8-epi-PGF_2α_ from platelets, ↑ NO upon collagen stimulation vs. controls [30]
Immunohistochemistry of NOX5 in carotid plaques	↑ NOX5 vs. non-atherosclerotic sections [31]
Immunohistochemistry of MPO in arteries from transplanted hearts	↑ MPO in the fibrous cap and lipid core vs. other lesion’s parts and normal arteries [32,33]

Abbreviations: ApoE: apolipoprotein E; CAD: cardiovascular disease; DHE: dihydroethidium; DCF: dichlorodihydrofluorescein; ECs: endothelial cells; EPR: electron paramagnetic resonance spectroscopy; HFD: high-fat diet; HUVECs: human umbilical vein endothelial cells; IL-1α: interleukin 1-alpha; IL-1β: interleukin 1-beta; I/R: ischemia reperfusion; LDLr: low-density lipoprotein receptor; L-012: luminol-based chemiluminescent probe; MDA: malondialdehyde; mRNA: messenger RNA; MPO: myeloperoxidase; NOXA-1: nicotinamide adenine dinucleotide phosphate oxidase activator-1; NOX: nicotinamide adenine dinucleotide phosphate oxidase; VCAM-1: vascular cell adhesion molecule 1; WT: wildtype; X-linked CGD: X-linked chronic granulomatous disease; XO: xanthine oxidase; ↑ indicates increase; ↓ indicated decrease.

**Table 2:** Scavenger systems and atherosclerosis in animal models and in human diseases.

Genetic Background and/or Experimental Setting	Phenotype
**Animal models**	
ApoE^−/−^/Cat^+/+^	↓ Plasma, aortic 8-epi-PGF_2α_, size and progression of atherosclerotic lesions [60] VCAM-1, ICAM-1, BaP-induced monocyte adhesion to ECs vs. ApoE^−/−^ [61]
Cat^+/+^ in SMCs	↓ MMP1, TNFα, apoptosis in aortas vs. WT [62]
LDLr^−/−^/mCat^+/+^	↓ MCP-1, Phosphorylation of RelA (NF-κB), macrophage infiltration into the atherosclerotic lesions, [63] ↓ Neutrophil extracellular traps and myeloid-cell accumulation in the atherosclerotic lesions vs. LDLr^−/−^ [64]
ApoE^−/−^/PRDX4^+/+^	↓ Ox-LDL levels in the plaques, CD3^+^ T cells, collagen in the fibrous caps, intimal lesions of the atherosclerotic aortic valves vs. ApoE^−/−^ [65]
PRDX1^−/−^	↑ Endothelial and soluble P-selectin, Von Willebrand factor vs. WT [66]
ApoE^−/−^/PRDX1^−/−^	↑ Macrophage infiltration into the atherosclerotic lesions and atherosclerotic lesion size vs. ApoE^−/−^ [66]
ApoE^−/−^/PRDX2^−/−^	↑ Activation of p65, c-Jun, JNKs, p38 MPK, VCAM-1, ICAM-1, MCP-1, TNFα in the plaques vs. ApoE^−/−^ [67]
ApoE^−/−^/GPX1^+/+^	↓ Aortic 8-epi-PGF_2α_, number and size of atherosclerotic lesions vs. ApoE^−/−^ [68]
GPX1^+/-^	↑ Plasma 8-epi-PGF_2__α_, perivascular matrix deposition vs. WT [69]
ApoE^−/−^/GPX1^−/−^	↑ Ox-LDL, macrophages infiltration, foam cells formation and proliferation, atherosclerotic lesions size, [70] VCAM-1, VEGF-1, p-63 activation, macrophages in aortas vs. ApoE^−/−^ [71]
Trx2^+/+^	↑ Total antioxidants and NO, ↓ Plasma 8-epi-PGF_2α_ in the atherosclerotic lesions vs. WT [72]
Trx2^−/−^	↑ ONOO^−^, arterial hypertrophy, vascular stiffness, apoptosis, fibrosis, ↓ NO vs. WT [73,74]
ApoE^−/−^/SOD1^+/+^	↓ 8-epi-PGF_2α_ in the plasma and aortas, size of atherosclerotic lesions vs. ApoE^−/−^ [60]
SOD1^−/−^	↑ O_2_^•−^ in the aorta assessed by LCD and susceptibility to experimental thrombosis vs. WT [75]
ApoE^−/−^/SOD2^+/-^	↑ 8-OHgua VCAM-1, Calpain-2, Caspase-3, MMP-2 in intimal VSMC, T-cell content and ↓ Collagen in the plaque vs. ApoE^−/−^ [76]
PON1^−/−^	↑ O_2_^•−^ in the aorta as assessed by LCD, VCAM-1, ICAM-1, P-selectin vs. WT [77]
ApoE^−/−^/PON1^+/+^	↓ Ox-LDL and atherosclerotic lesion size vs. ApoE^−/−^ [78]
LDL^−/−^/adenovirus-mediated PON1 gene transfer	↓ Ox-LDL levels in plasma and plaques vs. LDL^−/−^ [79]
ApoE^−/−^/PON2^−/−^	↑ O_2_^•−^ levels in the supernatants of aorta lysates, as assessed by DHE and atherosclerotic lesion size vs. ApoE^−/−^ [80]
ApoE^−/−^ injected with adenovirus PON2 (AdPON2)	↓ Ox-LDL and serum lipid hydroperoxides vs. ApoE^−/−^ [81]
**Human studies**	
Congenital Cat deficiency	↓ Cat levels, ↑ H_2_O_2_, atherosclerosis, and DM vs. subjects without acatalasemia [82,83,84]
599C/T allele of the GPX1 gene	↓ GPX activity, ox-LDL and ↑ MDA and risk of restenosis vs. non-carriers 599C/T allele [85]
Upregulation GPX1 in ECs in vitro	↓ CD40 protein, MCP-1 and VCAM-1 [86]
M/L54 PON1 polymorphisms	↓ Serum PON1 activity and ↑ CHD in carriers M/L54 PON1 DM patients vs. non-carrier DM patients [87]
M/L55 and Q/R 192 PON1 polymorphism	↓ Serum PON1 activity and ↑ CAD, carotid thickening and plaques in M/L54 and Q/R 192 PON1 carriers vs. non-carriers [88,89]
Immunofluorescence in carotid lesions	↓ PON2 expression in atherosclerotic lesions vs. healthy tissues [81]
Immunohistochemistry in coronary arteries	↑ Trx expression in VSMCs and macrophages of atherosclerotic vs. healthy coronary arteries [90]
Proteomics in aortic aneurysm tissues	↑ PRDX2 expression in patients with ruptured vs. non-ruptured aneurysms [91]
SOD3 _R213G_ polymorphism	↓ SOD3 activity and ↑ ischemic heart diseases [92]
T-allele of rs2284659 variant of SOD3 promoter	↑ SOD3 plasma levels and ↓ Circulating 8-epi-PGF_2α_, oxidation protein products, MI, in rs2284659 carriers DM patients vs. non-carrier DM patients [93]

Abbreviations: ApoE: apolipoprotein E; BAEC: bovine aortic; CAD: coronary artery disease; BaP: benzo(a)pyrene; DHE: dihydroethidium; DM: diabetes mellitus; ECs: endothelial cells; CAT: catalase; 8-epi-PGF_2α_: 8-epi-prostaglandin F_2α_; Q/R192: Gln-Arg; HDL: high-density lipoprotein; JNK: c-Jun N-terminal kinase; ICAM1: intercellular adhesion molecule 1; LDL: low-density lipoprotein; Ox-LDL: oxidized low-density lipoprotein; LCD: lucigenin-derived chemiluminescence; M/L54: met-Leu 54 PON1 polymorphism; M/L55: Met-Leu 55 PON1 polymorphism; mCat: mitochondrial catalase; MCP1: monocyte chemoattractant protein 1; MI: myocardial infarction; MMP-1: matrix metallopeptidase 1; MPK: mitogen-activated protein kinase; NF-κB: nuclear factor kappa light chain enhancer of activated B cells; 8-OHgua: 8-hydroxy-2′-deoxyguanosine; PRDX: peroxiredoxin; PON: paraoxonase; ROS: reactive oxygen species; SOD: superoxide dismutases; TNFα: tumor necrosis factor α; Trx: thioredoxin; VCAM: vascular cell adhesion molecule; VEGF: vascular–endothelial growth factor; VSMC: vascular smooth muscle cells; WT: wild type; ↑ indicates increase; ↓ indicates decrease.

**Table 3:** Oxidative stress biomarkers in ASCVD and related high-risk patients.

Study (Year)	Study Population	Design of the Study	Main Results
8-epi-PGF_2α_			
Davi et al. (1997) [125]	Hypercholesterolemic patients (*n* = 40) vs. matched controls (*n* = 40)	Cross-sectional study	8-epi-PGF_2α_: 473 ± 305 vs. 205 ± 95 pg/mg creatinine; *p* = 0.0001 in hypercholesterolemic patients vs. controls 8-epi-PGF_2α_ correlated with 11-dehydro-TXB_2_ in hypercholesterolemic patients, rho = 0.512; *p* = 0.0001
Davi et al. (2002) [124]	Healthy obese women (*n* = 44) vs. non obese matched controls (*n* = 24)	Cross-sectional study	8-epi-PGF_2α_: 523 (293–685) vs. 187 (140–225) pg/mg creatinine; *p* < 0.001 in obese women vs. controls 8-epi-PGF_2α_ correlated with 11-dehydro-TXB_2_ in obese women, rho = 0.61; *p* < 0.001
Keaney et al. (2003) [131]	Adult subjects (*n* = 2828)	Cohort study	8-epi-PGF_2α_: 240 ± 145 vs. 148 ± 100 ng/mmol creatinine; *p* < 0.0001 in smokers vs. non-smokers 8-epi-PGF_2α_: 181 ± 128 vs. 157 ± 108 ng/mmol creatinine; *p* < 0.0001 in DM vs. non-DM subjects 8-epi-PGF_2α_ independently significantly correlated with smoking, BMI, and history of CVD.
Schwedhelm et al. (2004) [132]	CAD patients (*n* = 93) vs. matched controls (*n* = 93)	Case-control study	8-epi-PGF_2α_: 139 (93–231) vs. 77 (61–101) pmol/mmol creatinine; *p* < 0.001 in CAD vs. controls 8-epi-PGF_2α_ correlated with 2,3-dinor-5,6-dihydro-8-iso-PGF_2α_, and CRP in CAD patients, rho = 0.225, *p* < 0.01, and rho = 0.321, *p* < 0.001, respectively 8-epi-PGF_2α_ correlated with DM, hypertension, smoking, hyperlipidemia, and BMI for all subjects; *p* < 0.001 for trend
Roest et al. (2008) [133]	Postmenopausal women (*n* = 12,239) including women who died of CHD (*n* = 141) and stroke (*n* = 109) vs. controls (*n* = 142)	Nested prospective case-cohort study Follow-up: 18 years	8-epi-PGF_2α_: 0.31 (0.23–0.46) vs. 0.23 (0.18–0.31) ng/mg creatinine; in smokers (*n* = 128) vs. non-smokers (*n* = 264), *p* < 0.001 CVD mortality risk higher for the highest of 8-iso PGF_2α_ vs. the lowest quartile, OR: 1.8 (95% CI; 1.1–3.1; *p* = 0.02)
Pascale et al. (2012) [129]	Patients with ET (*n* = 38)	Cross-sectional study.	8-epi-PGF_2α_ correlated with 11-dehydro-TXB_2_, rho = 0.55, *p* = 0.008
Zaccardi et al. (2016) [126]	T1DM patients (*n* = 51) vs. matched healthy controls (*n* = 63)	Cross-sectional study	8-epi-PGF_2α_: 796 ± 218 vs. 468 ± 235 pg/mg creatinine; *p* < 0.001 in T1DM patients vs. controls 8-epi-PGF_2α_ correlated with 11-dehydro-TXB_2_ in T1DM patients, rho = 0.75; *p* < 0.001
Petrucci et al. (2019) [123]	Healthy obese subjects (*n* = 19) vs. matched controls (*n* = 19)	Cross-sectional study	8-epi-PGF_2α_: 826 (129–549) vs. 555 (425–693) pg/mg creatinine; *p* = 0.03 in obese subjects vs. controls 8-epi-PGF_2α_ correlated with 11-dehydro-TXB_2_ in obese subjects, rho = 0.55; *p* = 0.02
Santilli et al. (2020) [128]	Subjects with IGT (*n* = 48), T2DM patients since <1 year (*n* = 60), and T2DM patients since >1 year (*n* = 58)	Cross-sectional study	8-epi-PGF_2α_: 594 (411–876) vs. 618 (402–1060) vs. 466 (371–716) pg/mg creatinine; *p* = 0.0138 in IGT subjects vs. new DM vs. established DM 8-epi-PGF_2α_ correlated with 11-dehydro-TXB_2_ in IGT and DM
**MDA**			
Noberasco et al. (1991) [134]	DM patients (*n* = 67) vs. matched healthy controls (*n* = 40)	Cross-sectional study	MDA: 3.69 ± 0.28 vs. 1.92 ± 0.13 nmol/mL; z = 4.48, α < 0.01 in DM patients vs. controls MDA is correlated with glycosylated hemoglobin in DM patients (rho = 0.29, α < 0.05)
Cavalca et al. (2001) [135]	CAD patients (*n* = 40) vs. matched healthy controls (*n* = 70)	Cross-sectional study	Total MDA: 2.6 (3.8–1.7) vs. 1.3 (2.2–0.9) µmol/L; *p* < 0.00001 in CAD patients vs. controls Free MDA: 0.5 (1.3–0.2) vs. 0.3 (0.7–0.05) µmol/L; *p* < 0.03 in unstable vs. stable angina group
Walter et al. (2004) [136]	CAD patients (*n* = 643)	Prospective cohort study Follow-up: 2 years	CAD patients in the highest vs. lowest quartile of MDA: MI (*n* = 51) RR: 2.94 (95% CI 1.75–4.94; *p* < 0.0001) Angina (*n* = 149) RR: 2.58 (95% CI 1.98–3.37; *p* < 0.0001) CABG/PTCA (*n* = 139) RR: 2.14 (95% CI 1.61–2.84; *p* < 0.0001)
Tanriverdi et al. (2006) [137]	Smokers (*n* = 36) vs. matched non-smokers controls (*n* = 51)	Cross-sectional study	MDA: 1.91 ± 1.3 vs. 1.18 ± 0.9 nmol/mL; *p* = 0.003 in smokers vs. controls SOD: 4267.7 ± 2842.8 vs. 2812 ± 665.4 U/gHb; *p* = 0.008 in smokers vs. controls GSH: 7.1 ± 1.8 vs. 8.5 ± 3.6 μmol/gHb; *p* = 0.019 in smokers vs. controls
Kotur-Stevuljevic et al. (2007) [138]	CAD (*n* = 141) vs. non-CAD controls (*n* = 47)	Cross-sectional study	MDA: 3.22 (1.336–7.762) vs. 2.66 (1.021–6.902) μmol/L; *p* < 0.001 in CAD patients vs. controls MDA in CAD patients independently correlated with fibrinogen and CRP: β = 0.262; *p* < 0.01and β = 0.331; *p* < 0.001, respectively
Kubihal et al. (2019) [139]	Healthy smokers (*n* = 75) vs. matched non-smokers controls (*n* = 25)	Cross-sectional study	MDA: 5.15 ± 0.39 vs. 4.11 ± 0.55 nmol/mL; *p* < 0.0001 in smokers vs. controls Vitamin C: 10.35 ± 1.44 vs. 13.9 ± 1.45 mg/L; *p* < 0.0001 in smokers vs. controls
**Ox-LDL**			
Ehara et al. (2001) [140]	Patients with acute MI (*n* = 45) vs. matched healthy controls (*n* = 46)	Cross-sectional study	Ox-LDL: 1.95 ± 1.42 vs. 0.58 ± 0.23 ng/5µg LDL; *p* < 0.0001 in patients with MI vs. controls
Shimada et al. (2004) [141]	CAD patients (*n* = 238) with (*n* = 162) vs. without cardiac events controls (*n* = 76)	Prospective cohort study Follow-up: over 4 years	Ox-LDL: 20.3 (17.5–30) vs. 17.6 (13.2–24.7) U/mL; *p* = 0.002 in patients with events vs. controls Cardiac event risk in patients in the highest vs. lowest quartile of ox-LDL, HR: 3.15 (95% CI 1.47–6.76; *p* = 0.003)
Tsimikas et al. (2006) [142]	Men and women aged 40-80 years (*n* = 826)	Prospective study Follow-up: 5 years	Ox-LDL circulating levels associated with the incidence and progression of carotid atherosclerosis, β = 0.17; *p* = 0.001, OR: 1.44 (95% CI 1.06–1.96; *p* = 0.02) and femoral atherosclerosis, *β* = 0.16; *p* = 0.003, RR: 1.34 (95% CI 1.05–1.71; *p* = 0.018)
Zhang et al. (2014) [143]	ACS patients (*n* = 425)	Prospective cohort study Median follow-up: 30 months	Ox-LDL: 283.22 ± 38.93 vs. 198.62 ± 56.42 mmol/L; *p* < 0.01 in event vs. event free patients hsCRP: 20.75 ± 5.37 vs. 14.22 ± 4.18 mg/L; *p* < 0.01 in patients with or without events Ox-LDL and hsCRP correlated rho = 0.67, *p* < 0.01
Gao et al. (2017) [144]	Adults with vs. without CVD (*n* = 8644)	Meta-analysis of 12 observational studies	Summary effect size of increased circulating ox-LDL was 1.79 (95% CI 1.56–2.05) for ASCVD. There was no statistical heterogeneity observed across studies (Q = 15.22; *p* = 0.230; *I*^2^ = 21.2%)
**Nitrotyrosine**			
Ceriello et al. (2001) [145]	T2DM patients (*n* = 40) vs. matched healthy controls (*n* = 35)	Cross-sectional study	Nitrotyrosine: 0.251 ± 0.141 µmol/L vs. <10 nmol/L in T2DM patients vs. healthy controls Nitrotyrosine correlated with plasma glucose concentration in T2DM patients, rho = 0.38; *p* < 0.02
Shishehbor et al. (2003) [146]	Patients with CAD (*n* = 100) PAD (*n* = 36) vs. non-CAD controls (*n* = 108)	Cross-sectional study	Nitrotyrosine: 9.1 (4.8–13.8) vs. 5.2 (2.2–8.4) μmol/mol tyrosine; *p* < 0.001 in CAD patients vs. controls; 9.6 vs. 5.7 μmol/mol tyrosine; *p* = 0.001 in CAD patients with DM vs. non-DM patients. CAD risk in the upper vs. lower quartile in CAD patients without PAD, OR: 4.4 (95% CI 1.8–10.6; *p* < 0.001) CAD in the upper vs. lower quartile in CAD patients with PAD, OR: 26.3 (95% CI 2.9–238; *p* < 0.001) Atherosclerosis prevalence: 46% vs. 3%; *p* < 0.001 in CAD plus PAD patients in the highest quartile of nitrotyrosine vs. lowest quartile
**Protein carbonyl**			
Kilhovd et al. (1999) [147]	T2DM patients (*n* = 53, vs. matched non-DM subjects (*n* = 34)	Cross-sectional study	AGEs: 7.4 (4.4–10.9) vs. 4.2 (1.6–6.4) U/mL; *p* < 0.0001 in T2DM patients vs. controls; 8.1 [6,4,5,6,7,8,9,10,9] vs. 7.1 (3.5–9.8) U/mL, *p* = 0.03 in T2DM with CHD vs. without CHD AGEs associated with CHD in T2DM patients, OR: 2.4 (95% CI 1.2–4.8; *p* = 0.008)
De Cristofaro et al. (2003) [148]	T2DM patients (*n* = 72) vs. matched healthy controls (*n* = 72)	Cross-sectional study	Protein carbonyls: 6.1 ± 1.4 vs. 4.6 ± 1 × 10^−6^ *w*/*w*; *p* < 0.05 in T2DM patients vs. controls Protein carbonyls correlated with 8-epi-PGF_2α_ in T2DM patients, rho = 0.242; *p* = 0.039
Mutlu-Türkoglu et al. (2005) [149]	CAD patients (*n* = 30) vs. matched healthy controls (*n* = 30)	Cross-sectional study	Protein carbonyls: 1.1 ± 0.05 vs. 0.9 ± 0.02 nmol/mg protein, *p* < 0.01 in CAD patients vs. controls
Semba et al. (2009) [150]	Dwelling women, aged ≥65 years (*n* = 559)	Prospective study Follow-up: 4.5 years	CVD mortality in dwelling women (*n* = 54), CVD in subjects in the highest quartile of AGEs: HR 2.29 (95% CI, 1.21–4.34; *p* = 0.01)
Pirinccioglu et al. (2010) [151]	Hypercholesteraemic patients (*n* = 25) vs. matched healthy controls (*n* = 25)	Cross-sectional study	Protein carbonyls: 2.12 ± 0.26 vs. 1.52 ± 0.28 nmol/mg protein; *p* < 0.001 in hypercholesteraemic patients vs. controls Protein carbonyls are correlated with MDA and IMT in hypercholesterolemic patients, rho = 0.77; *p* < 0.001, and rho = 0.82; *p* < 0.001, respectively
Vegi et.al (2012) [152]	T2DM patients (*n* = 60) vs. matched healthy controls (*n* = 60)	Cross-sectional study	Protein carbonyls: 1.68 ± 0.47 vs. 0.7 ± 0.34 nmol/L; *p* < 0.001 in T2DM patients vs. controls
Van Eupen et al. (2013) ([153]	T1DM patients (*n* = 165) vs. matched non-DM controls (*n* = 169)	Cross-sectional study	Plasma levels in protein- bound N^ε^-(carboxymethyl) lysine: 105 (102–107) vs. 93 (90–95) nmol/mmol LYS; *p* < 0.001 in T1DM patients vs. controls Plasma levels in protein-bound Pentosidine: 0.69 (0.65-0.73) vs. 0.51 (0.48-0.54) nmol/mmol LYS; *p* < 0.001 in T1DM patients vs. controls Plasma levels in protein-bound Pentosidine: 0.81 [0.70–0.93] vs. 0.67 (0.63–0.71) nmol/mmol LYS; *p* = 0.028 in T1DM patients with moderate to high CAC vs. low CAC score
McNair et al. (2016) [154]	Hypercholesterolemic ACS patients (*n* = 55) vs. matched normocholesterolemic ACS controls (*n* = 45)	Cross-sectional study	AGEs: 1213 ± 68.6 vs. 642 ± 22 ng/mL, *p* = 0.001 in hypercholesterolemic patients vs. controls AGE/sRAGE ratio: 1.71 ± 0.16 vs. 0.49 ± 0.02; *p* < 0.001 in hypercholesterolemic patients vs. controls AGEs are correlated with total cholesterol, LDL-C, and triglycerides, rho = 0.664, rho = 0.66, and rho = 0.741; *p* < 0.001, respectively
Kopytek et al. (2020) [155]	T2DM patients with atherosclerosis (*n* = 50) vs. matched non-DM with atherosclerosis controls (*n* = 76)	Cross-sectional study	AGEs: 9.55 (8.56–10.92) vs. 0.73 (0.68–0.77) ng/mL; *p* < 0.0001 in T2DM patients with atherosclerosis vs. non-DM with atherosclerosis Valvular AGEs in all DM patients are associated with AVA rho = 0.68; *p* < 0.0001
Sharifi-Zahabi et al. (2021) [156]	Adults with and without DM and CVD (*n* = 3718)	Systematic review and meta-analysis of Prospective Observational Studies	AGEs associated with increased risk of the following: all-cause mortality (pooled effect measure: 1.05; 95% CI: 1.01, 1.09; *p* = 0.018), and CVD mortality (pooled effect measure: 1.08; 95% CI: 1.01, 1.14; *p* = 0.015)

Results are presented as mean ± standard deviation or median and [interquartile range], as appropriate. Abbreviations: ACS: acute coronary syndrome; AGEs: advanced glycation end products; ASCVD: atherosclerotic cardiovascular disease; AVA: aortic valve area; β: regression coefficient; CABG: coronary artery bypass grafting; CAC: coronary artery calcification; CAD: coronary artery disease; CHD: coronary heart disease; CI: confidence interval; hsCRP: high-sensitivity C-reactive protein; CVD: cardiovascular disease; CV: cardiovascular; DM: diabetes mellitus; 8-epi-PGF_2α_: 8-epi-prostaglandin F_2α_; ET: essential thrombocythemia; GSH: glutathione; HR: hazard ratio; IMT: intima-media thickness; LDL-C: low-density lipoprotein cholesterol; LYS: lysine; MDA: malondialdehyde; MI: myocardial infarction; OR: odd ratio; Ox-LDL: oxidized low-density lipoprotein; PTCA: percutaneous transluminal coronary angioplasty; RR: relative risk; PAD: peripheral artery disease; PTCA: percutaneous transluminal coronary angioplasty; SOD: superoxide dismutase; T1DM; type 1 diabetes mellitus T2DM: type 2 diabetes mellitus; TXB_2_: thromboxane B_2_.
